# Controlled creation and displacement of charged domain walls in ferroelectric thin films

**DOI:** 10.1038/srep31323

**Published:** 2016-08-10

**Authors:** L. Feigl, T. Sluka, L. J. McGilly, A. Crassous, C. S. Sandu, N. Setter

**Affiliations:** 1 Ceramics Laboratory, EPFL – Swiss Federal Institute of Technology, Lausanne, CH-1015 Switzerland; 2 Institute for Photon Science and Synchrotron Radiation, KIT - Karlsruhe Institute of Technology, Hermann-von-Helmholtz-Platz 1, D-76344 Eggenstein-Leopoldshafen, Germany

## Abstract

Charged domain walls in ferroelectric materials are of high interest due to their potential use in nanoelectronic devices. While previous approaches have utilized complex scanning probe techniques or frustrative poling here we show the creation of charged domain walls in ferroelectric thin films during simple polarization switching using either a conductive probe tip or patterned top electrodes. We demonstrate that ferroelectric switching is accompanied - without exception - by the appearance of charged domain walls and that these walls can be displaced and erased reliably. We ascertain from a combination of scanning probe microscopy, transmission electron microscopy and phase field simulations that creation of charged domain walls is a by-product of, and as such is always coupled to, ferroelectric switching. This is due to the (110) orientation of the tetragonal (Pb,Sr)TiO_3_ thin films and the crucial role played by the limited conduction of the LSMO bottom electrode layer used in this study. This work highlights that charged domain walls, far from being exotic, unstable structures, as might have been assumed previously, can be robust, stable easily-controlled features in ferroelectric thin films.

Domain walls in ferroelectric materials can exhibit unique properties, different from the bulk material, due to the local change in strain, charge or symmetry^1^,^2^. Thereby they form mobile homo-interfaces within the material in contrast to hetero-interfaces which have a fixed position. Focus has recently shifted towards the properties of domain walls, in particular their conductivity^3^,^4^,^5^,^6^, in the hope of utilizing them as elements in envisioned ‘nanoelectronic’ structures^7^. As a result extensive work has been undertaken to control the type, period and arrangement of domain walls in ferroelectric thin films through processing conditions and materials choices^6^,^8^,^9^,^10^,^11^. Most of the work has been directed towards electrostatically neutral domain walls. It is now emerging that the special case of violation of the electrostatic neutrality, necessarily causing formation of ‘charged domain walls’ (CDW), leads to exciting property enhancement^12^. This has been demonstrated both in epitaxial (001) oriented thin films^13^,^14^,^15^ and (110) oriented single crystals^16^ wherein CDWs are usually associated with strong local electrical conduction.

The creation and control of CDWs remains a challenge. Traditionally, CDWs are assumed to be energetically highly unfavorable and as such suitably difficult to create. It has been shown recently that under special circumstances, involving polarization switching, CDWs can be created but these techniques tend to be complex and time consuming^14^,^16^,^17^. A simple, reliable and robust method to create CDW in thin film architectures is desired; here we show exactly such a possibility.

We use epitaxial (110) oriented (Pb,Sr)TiO_3_ (PST) thin films for the demonstration of the concept. PST is a practical choice due to the good lattice match with SrTiO_3_ 18,19 (STO) and the significantly better quality of our in-house grown (110) oriented films compared to previously attempted (110) BaTiO_3_ films. Like the case of tetragonal BTO single crystal^16^, applying an electric field along [110] of the tetragonal PST thin film is a prerequisite for the creation of CDW. The small tetragonal distortion of PST^20^ and a small compressive mismatch with STO impose only weak mechanical restrictions, even in the (110) plane. At the same time, the remnant polarization is comparable to BaTiO_3_ 21,22 providing sufficient charge to create strongly charged domain walls. We first demonstrate by means of piezoresponse force microscopy (PFM) the creation of CDWs in (110) oriented tetragonal ferroelectric PST thin films during 180° polarization switching. The main advantage of this method is its simplicity. Furthermore, as the sole requirement for CDW formation is switching the out-of-plane component of the polarization, we are able to extend the possibility to create, move and annihilate such walls using a solid-state top electrode hence bringing these homo-interfaces one step closer to devices.

## Results and Discussion

**Figure 1:**
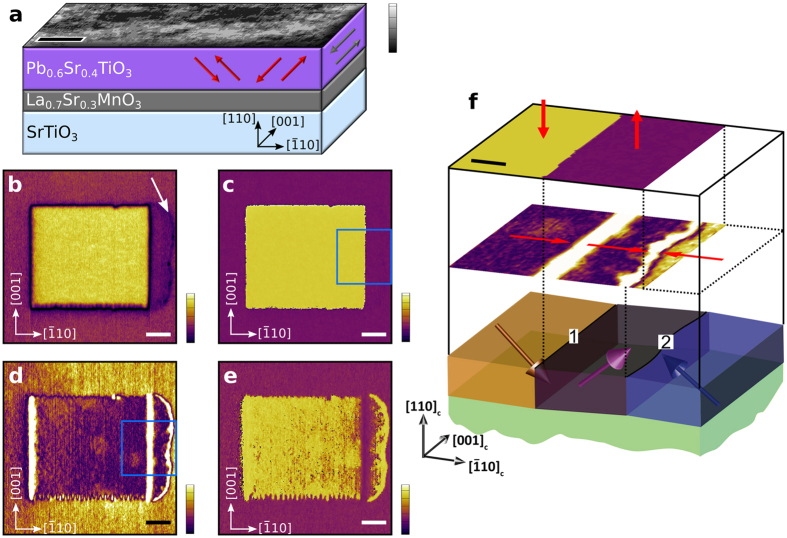
(**a**) AFM topography image of PST top layer (z-scale is 0.5 nm, scale bar is 500 nm) together with a schematic of the PST/LSMO//STO(110) multilayer. Possible ferroelectric polarization directions are represented by the red arrows. Polarization along [001] (greyed out) is suppressed by compressive strain. *Vertical* PFM (**b**) amplitude and (**c**) phase images after switching a 2.5 × 2.5 μm^2^ region downwards. The corresponding *lateral* PFM (**d**) amplitude and (**e**) phase images. Scale bars are 500 nm. Vertical scale is in a.u. for PFM amplitude and 360° for PFM phase. (**f**) Close-ups of the PFM signal (top two planes) from the regions marked by the blue squares in (**c**) and (**d**), respectively, and schematic of resulting domain structure (bottom). Blue indicates the initial state, yellow the region switched by 180° and purple the region where only the lateral component was switched. Polarization components are indicated by plain red arrows and polarizations by the 3D arrows. The neutral 90° domain wall is indicated by 1, and charged domain wall by 2. Scale bar is 100 nm.

The topography of a PST (110) thin film is shown in Fig. 1a together with a schematic representation of the sample geometry and the crystallographic relationship of the epitaxial layers. The (110) plane STO substrate imposes the same orientation to the LSMO electrode and the PST ferroelectric thin film. Generally, three polarization directions, each with two opposite senses, are possible in tetragonal ferroelectrics (indicated by arrows in Fig. 1a). However, due to the anisotropic biaxial compressive strain exerted by the STO substrate, in-plane polarization along the indicated [001] or [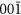] direction (Fig. 1a) is highly unfavorable^23^ and consequently not observed experimentally. Since no relaxation takes place, both misfit and threading dislocations are absent. This very low defect density results in films with a very low roughness (Fig. 1a, RMS ~0.1 nm) allowing for unobstructed observation of domain structure.

Both vertical (Fig. 1b,c) and lateral (Fig. 1d,e) PFM amplitude and phase signals of the PST thin film are recorded after having switched a 2.5 × 2.5 μm^2^ rectangular area. Whereas the vertical phase (Fig. 1c) shows a pure 180° change of the switched area an additional contrast is visible in the vertical amplitude (Fig. 1b, indicated by a white arrow). The lateral PFM (Fig. 1d,e) confirms the 180° phase change of the switched area but also reveals an additional pure in-plane polarization change in an area outside the switched region. We hereafter call this region a ‘secondary domain’.

Regarding the interpretation of the lateral PFM data it has to be noted that the border between purple and yellow region inside the secondary domain in Fig. 1e is noisy and differs from scan to scan which is a typical indication of cross-talk with the vertical phase signal^24^,^25^,^26^. Moreover, the lateral PFM amplitude, Fig. 1d, shows the same magnitude in the switched primary domain and the secondary domain, suggesting the same in-plane response and therefore the same orientation of in-plane polarization component for both domains. Additionally, the opposite response of the domains in case of opposite out-of-plane polarization components causes a buckling of the cantilever which appears as a fake in-plane piezoelectric response as seen by bright lines at 180° and neutral 90° domain walls^27^. Regarding the strong piezoelectric response at the charged domain wall, various artifacts at DWs are rather common in the lateral PFM mode. We can only speculate whether the strong response arises from electrostatic charges accumulated during scanning, extrinsic contributions due to tiny domain wall motion, real enhanced lateral piezoelectric response at these DWs, or due to the inherent error in the method involving shift and change of tip/contact resonance mode. Apart from that, turning the sample by 90° causes a vanishing of the lateral PFM signal (see Supplementary Fig. S1) confirming the restriction of the polarization to the [100] and [010] directions.

Combining the PFM data with the known sample orientation and polarity of the applied switching bias allows us to reconstruct the domain structure (Fig. 1f). The switched rectangle changed both the in-plane and the out-of-plane phase and therefore has polarization opposite to the as-grown film. The secondary domain changed only the in-plane phase and its polarization therefore points up and against the original in-plane state. This creates a 90° DW between the primary and the secondary domain (1 in Fig. 1f) and inevitably also a head-to-head CDW between the outer boundary of the secondary domain and the as-grown domain (2 in Fig. 1f).

**Figure 2:**
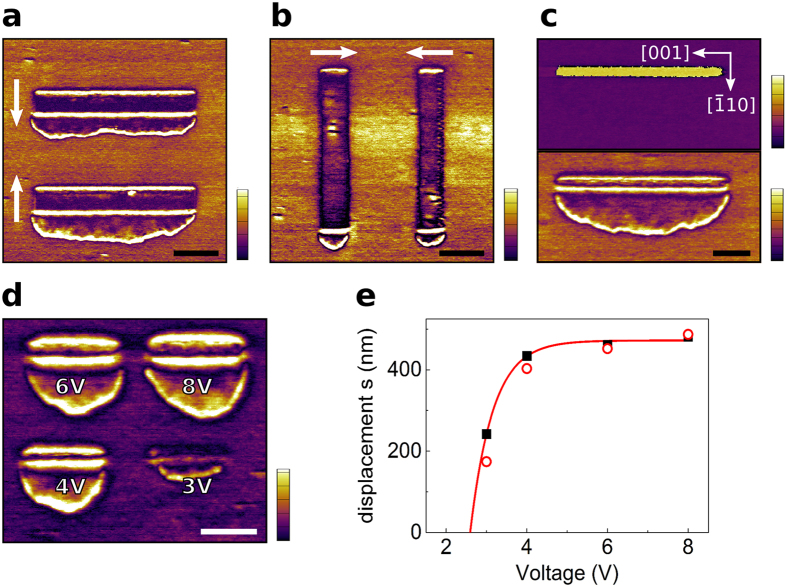
(**a**–**c**) PFM images of PST ferroelectric layer, scale bars are 1 μm, vertical scale is in a.u. for PFM amplitude and 360° for PFM phase, crystallographic directions displayed in (**c**) are representative for all PFM images. (**a**,**b**) Lateral PFM amplitude images of poled regions, different slow scan directions of the tip are represented by white arrows. The secondary domain always appears at the same side, towards the [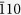] direction. (**c**) Vertical PFM phase (top) and lateral PFM amplitude (bottom) images of a switched line, switching was done at a scan speed of 100 nm/s. (**d**) Lateral PFM amplitude image of regions poled with different voltages as shown in the image. Scale bar is 500 nm. (**e**) Measured (squares and dots) and extrapolated (full line) displacement of the CDW as a function of the applied switching voltage.

More detailed switching experiments were done in order to closer investigate the formation mechanism of the secondary domain. As can be seen from the lateral amplitude (Fig. 2a,b) the position of the secondary domain is strictly limited to one side of the switched area, towards the [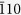] direction. This position was neither affected by changing the slow scan direction (indicated by arrows) to create a trailing field effect^14^,^28^, nor by the orientation of the switched area. Also the size of the switched area doesn’t influence the size of the secondary domain which is created even in the case of switching along one single line (Fig. 2c). Increasing the voltage while scanning with the same speed results in a further displacement of the CDW (Fig. 2d). At 2 V no switching could be observed. This agrees well with the extrapolated displacement of the CDW, *s*, with respect to the applied voltage where no displacement is expected below 2.6 V (Fig. 2e).

**Figure 3:**
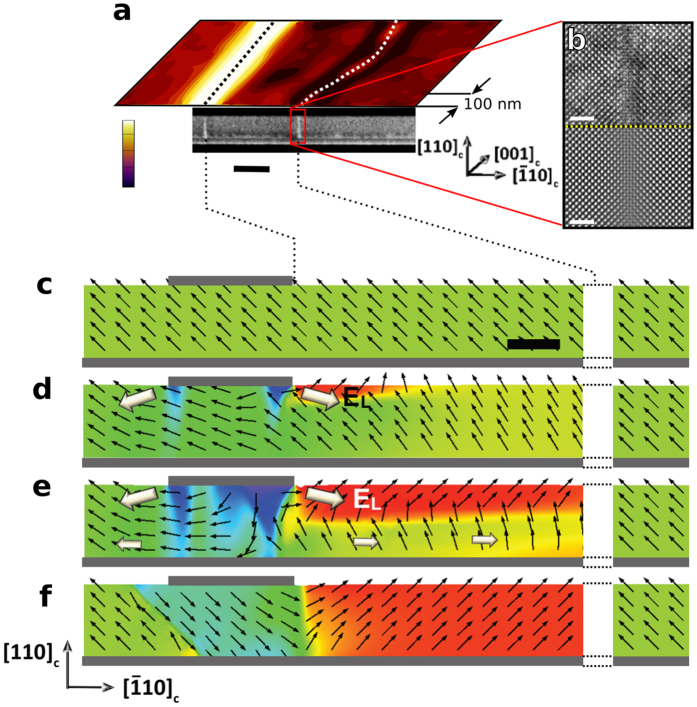
(**a**) Lateral PFM amplitude and the corresponding TEM image of a poled region in the PST ferroelectric layer, both set to the same scale. Scale bar is 100 nm, vertical scale of PFM amplitude in a.u. Domain walls are indicated by dotted lines. (**b**) HRTEM (upper panel) and corresponding Fourier filtered image (lower panel) of the CDW present between the secondary domain and the non-switched area. Scale bar is 2 nm. (**c**–**f**) Phase field simulation of the switching process. Scale bar is 40 nm. The black arrows represent the local ferroelectric polarization direction. The simulation spans only a small area, farther away on the right would be the original polarization direction again. (**c**) As grown single domain state. (**d**) The applied dc bias voltage between the top and bottom electrodes (gray lines) initiates 180° switching under the electrode and nucleates side domain by in-plane 90° switching on the side of the top electrode. (**e**) The in-plane electric field due to the limited conductivity of LSMO and the charge transfer propagates the secondary domain. (**f**) Completed stable domain structure after the dc bias voltage is removed.

With the aim of revealing the cross-sectional view of domain walls, an area was switched by PFM and a FIB cut was performed in the (001) plane to obtain a perpendicular section through the domain walls. Figure 3a shows a close-up of the PFM amplitude image with the corresponding scanning TEM (STEM) cross-sectional image where the DWs observed in STEM are correlated with the PFM. High Resolution TEM (HRTEM) and the corresponding filtered image of the same region are shown in Fig. 3b. They reveal a strongly distorted region of about 1 nm width due to elastic strain evidencing a ferroelastic domain wall. A more in-depth analysis of the structural details by HRSTEM is in progress but beyond the scope of this study.

Figure 3c–f shows the switching process obtained by phase field simulation (details in Methods) wherein the field is applied between the small top electrode and the continuous bottom electrode mimicking the situation found in PFM or under a narrow electrode. Since no reliable phase field parameters for PST are available at the moment, we demonstrate the fundamental switching mechanism on well tested model of prototypical ferroelectric BaTiO_3_. The details in switching process may differ between the two materials, but the qualitative features of side domain nucleation is clearly robust which we tested by variation of simulation parameters such as temperature and electrostriction coefficients. A lateral electric field component, *E*_L_, at the electrode edges induces nucleation of the secondary domain which inevitably faces the as-grown state in a head-to-head arrangement. The simulation assumes purely dielectric material and therefore cannot result in free carrier compensated CDWs but still demonstrates the principle of the in-plane switching process. The lateral electric field, which must be present to create and displace the observed CDWs, will always appear in the vicinity of a PFM tip or electrode edge. However, as this field is highly inhomogeneous, it would rapidly decrease in the lateral direction and is usually negligible around distances corresponding to the film thickness. As the observed secondary domain is much larger (~500 nm) than the film thickness (70 nm) this excludes the field inhomogeneity at the PFM tip or the electrode edges as the only contribution to the CDW formation. In fact the secondary domain propagates to a larger distance only if a high resistivity bottom electrode is taken into account, where slowly redistributing electric charges create an in-plane potential drop during switching, as is the case for the phase field simulations presented here (Fig. 3d–f).

The mechanism demonstrated both experimentally and by the simulation can be understood as follows: Switching an area of the as-grown single domain film by 180° flips the polarization downwards thereby pushing the positive polarization charge to the bottom electrode. The switched polarization charge needs to be compensated by charge carriers transported through the LSMO electrode. However, the resistivity of high quality (001) plane LSMO is reported to be as large as 1000 μΩ cm at room temperature^29^, orders of magnitude higher than good metals (Pt: 10 μΩ cm, Au: 2 μΩ cm) or the commonly used SrRuO_3_ (150 μΩ cm)^30^. Four-point probe measurements on different (110) plane LSMO thin films used here, with a thickness of 25 nm, reveal a resistivity of 5900 ± 250 μΩ cm along the [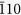] direction and 12000 ± 250 μΩ cm along [001]. The generally higher resistivity of the (110) plane LSMO can be attributed to the fact that it is not possible to achieve a single terminated (110) plane STO substrate which results in a more defective interface causing stronger electron scattering. The even higher resistivity observed along the [001] direction does not directly affect the switching process since no component of the polarization is lying in this direction. Owing to the high resistivity, an electric current due to the electrons transported through the LSMO creates the in-plane electric field *E*_L_, as illustrated in Fig. 3d,e, which supports the side domain propagation in the [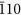] direction. On the opposite side of the switched area the local field in [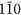] is created as well but just supports the already existing polarization state. Since the formation of the secondary domain appears only on one side of the switched area this allows determination of the lateral sense of direction of the as-grown in-plane polarization component, pointing along [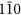] as already presented in the schematics.

**Figure 4:**
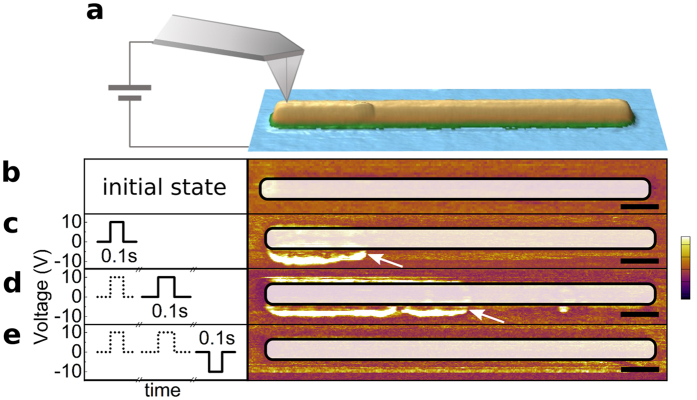
(**a**) AFM topography image of a Pt electrode with dimensions of l × w × h = 5000 × 250 × 4 nm^3^ deposited on the PST film and representation of the tip position for the switching procedure. (**b**–**e**) Application of switching pulses on the top electrode (left) and the resulting lateral PFM amplitude images (right). Scale bars are 500 nm, vertical scale of PFM amplitude in a.u. (**b**) The film exhibits a homogeneous polarization in the initial state. (**c**) Due to 180° switching below the electrode, a secondary domain is created (white arrow). (**d**) Repeating the same pulse causes the secondary domain to move further. (**e**) Application of opposite voltage resets original state, the secondary domain is fully erased.

The understanding of the mechanism involved in the creation of the secondary domain offers us the possibility to control its behavior. The strict connection of the CDW to the switched area was confirmed by PFM on the bare surface, causing the CDW to move or disappear if the switched area is changed or removed, respectively (see Supplementary Fig. S2). In order not to depend on the PFM tip for the creation of the CDW, low conductivity Pt line electrodes were prepared by electron beam induced deposition from a precursor gas, which allows control of the length of the switched region^31^,^32^. As added schematically to the actual topography image in Fig. 4a, the PFM tip was placed on one side of the electrode and only used to locally apply the voltage, without any scanning. A train of pulses of 0.1 s (Fig. 4c–e) was used to successively create, move and annihilate a CDW. The corresponding lateral PFM amplitude is shown in Fig. 4b–e where the superposed frame indicates the electrode area. Starting from the initial single domain state (Fig. 4b) a short voltage pulse is applied causing the area below the electrode to switch and the accompanying CDW to appear (Fig. 4c, indicated by arrow). An additional pulse of the same polarity causes a progression of the CDW (Fig. 4d) whereas a pulse of opposite polarity enables a reset of the structure to its initial state (Fig. 4e). This demonstrates the reproducibility and reversibility of this process offering a practical method to control the CDW.

The most intriguing property of CDWs presented in several previously published works^5^,^6^,^13^,^14^,^16^ is the CDW conductivity. We therefore scanned CDW regions with conductive AFM. However, enhanced conductivity was not found. Here it is necessary to emphasize, that conductivity of CDWs is not a guaranteed property. It is well established today that CDW conductivity is controlled by several factors beside the intrinsic conductivity. For instance, the contact barriers in reverse bias and associated contact resistances can in principle prevent entirely observation of conduction even if the CDW itself is conducting^16^. Moreover, even intrinsic conductivity of CDWs is not guaranteed. First principles calculations on similar compound PbTiO_3_ 33 show that, with increasing charging, CDWs will experience band splitting and pass through conducting state attaining finally again to an insulating state when they are strongly charged. Also defect compensation, which should not play an important role at room temperature, but which in principle suppresses CDW conductivity, cannot be entirely excluded. Last, but not least, CDWs can always acquire a shape which does not provide complete conductive path between electrodes. Evidently, the possible reasons for not having directly observed enhanced conduction are manifold. Identification of the exact cause is a new challenge which however exceeds the scope of this paper. On the other hand, this doesn’t limit the validity of the described mechanism which represents an alternative, convenient method to create charged domain walls.

## Summary

We have shown by the combination of vertical and lateral PFM data that switching (110) oriented epitaxial tetragonal ferroelectric thin films results in the creation of a secondary domain accompanied by a charged domain wall. This is attributed to the inhomogeneity of the electric field at sharp electrode edges in combination with the low conductivity of the bottom electrode. Both effects together cause the build-up of a lateral electric field able to switch and propagate the in-plane polarization component of the weakly tetragonal material, hence forming a charged domain wall. Furthermore, our investigations evidenced that the creation and dimension of the charged domain wall are determined by the characteristics of the applied voltage during the 180° switching of the film. Combining this with a low conductivity top electrode allows for a precise control of the position of the switched area and simultaneously of the charged domain wall. Moreover, the presented system is based on STO as a substrate material which can be grown as a buffer layer on Si and therefore might allow the realization of these structures on Si platform as well. These results represent a further step in the ongoing effort to precisely tune domain structures as building blocks of envisioned ‘nanoelectronics’.

## Methods

Thin films were fabricated by pulsed laser deposition on (110) plane STO substrates (Crystec GmbH, Germany) after etching in buffered HF and annealing at 900 °C in air. Using a laser fluence of ~1 J/cm^2^, conditions for growth of the bottom electrode La_0.67_Sr_0.33_MnO_3_ (LSMO) layer and the ferroelectric Pb_0.6_Sr_0.4_TiO_3_ layer were T_S_ = 625 °C, p_O2_ = 0.14 mbar, f = 2 Hz and T_S_ = 575 °C, p_O2_ = 0.25 mbar, f = 3 Hz, respectively.

PFM was performed on an Asylum Research Cypher with Ti/Ir coated ASYELEC-01 silicon probes with a force constant of 2 N/m.

Focused ion beam (FIB) cutting was conducted with a ZEISS NVision using a 30 kV acceleration voltage and beam current from 700 pA down to 40 pA after having deposited a protective carbon coating of 1 μm.

Transmission electron microscopy (TEM) was carried out with a Tecnai Osiris instrument using an acceleration voltage of 200 kV.

The two-dimensional phase field model (results in Fig. 3) was adopted from ref. 16 and modified as follows. Since Landau energy coefficients of PST are not known, the original coefficients of (110) tetragonal BaTiO_3_ from ref. 16, but at temperature 353 K, where tetragonality is smaller due to proximity to the paraelectric phase, were used. The free charge in the bulk of the 60 nm × 2.1 μm rectangular film is excluded, i.e. the material is assumed to be an ideal dielectric. The top (110) surface is mechanically free and has a 100 nm long ideal electrode where a 70 ns electric potential pulse with smooth edges is applied. The rest of the top surface contains fixed screening charge that fully compensates the charge of the [110] component of the spontaneous polarization. The bottom boundary is clamped to a rigid plane without a strain mismatch. Full bottom electrode is assumed as a non-ideal one dimensional conductor with defined electron drift and diffusion. The electron concentration was chosen as 10^19^ cm^−3^ and mobility was varied in order to mimic the effect of the limited conductivity. When the mobility is <10^−24^ cm^2^/(Vs) (note that mobility must be considered relatively to the simulation time) the secondary domain always propagates to a distance larger than the film thickness. Zero potential and constant electrode charge is defined 1 μm from the switching event. Note that the 50–100 nm long secondary domain appears always even in the case with an ideal bottom electrode, but it retracts and disappears after the switching event. In reality it could be pinned and survive, i.e. the secondary domain could be possibly observed even when using high quality bottom electrodes.

## Additional Information

**How to cite this article**: Feigl, L. *et al*. Controlled creation and displacement of charged domain walls in ferroelectric thin films. *Sci. Rep*. **6**, 31323; doi: 10.1038/srep31323 (2016).

## Supplementary Material

Supplementary Information
