# Supplementary material for: Controlled creation and displacement of charged domain walls in ferroelectric thin films
Source: Sci Rep. 2016 Aug 10;6:31323. doi: 10.1038/srep31323 (PMC4979207; doi:10.1038/srep31323)
Supplement: Supplementary Information [file srep31323-s1.pdf]

Controlled creation and motion of charged domain walls in ferroelectric thin films: Supplementary Information

L. Feigl, T. Sluka, L. J. McGilly, A. Crassous, C. S. Sandu, N. Setter

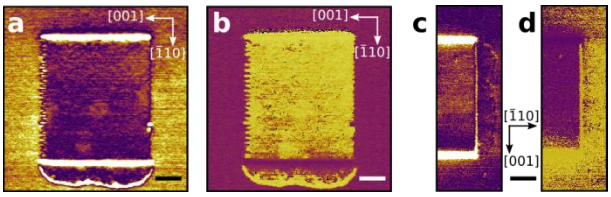

Supplementary Fig. S1: Lateral PFM amplitude (a and c) and phase (b and d) measuring the PFM signal along the [$\bar{1}$10] (a-b) and along the [001] (c-d) direction by turning the sample by 90°. Scale bars are 500 nm. The lateral phase contrast vanishes along the [001] direction, evidencing that the in-plane component of the polarization is lying in the [$\bar{1}$10] direction.

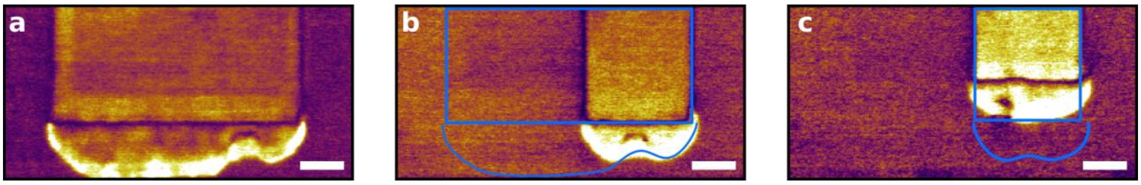

Supplementary Fig. S2: Lateral PFM amplitude illustrating the modification of the CDW by 180° switching. (a) shows the freshly switched rectangular area and the accompanying CDW. (b) If part of the initial area is switched back to the original state, the corresponding CDW disappears (blue lines indicate the preceding state). (c) If the size of the switched area is reduced, the CDW is drawn back as well (blue lines indicate the preceding state). Scale bars are 500 nm.
